# Structural Characterization and Antioxidant Potential of Chitosan by γ-Irradiation from the Carapace of Horseshoe Crab

**DOI:** 10.3390/polym12102361

**Published:** 2020-10-15

**Authors:** Siddhartha Pati, Anil Chatterji, Bisnu Prasad Dash, Bryan Raveen Nelson, Tanmay Sarkar, Salwa Shahimi, Hisham Atan Edinur, Teh Sabariah Binti Abd Manan, Paramananda Jena, Yugal Kishore Mohanta, Diptikanta Acharya

**Affiliations:** 1 Horseshoe Crab Research Unit, Department of Bioscience & Biotechnology, Fakir Mohan University, Balasore 756089, Odisha, India; bisnubsbtfmu@gmail.com; 2 Institute of Tropical Biodiversity and Sustainable Development, University Malaysia Terengganu, Kuala Nerus 21030, Terengganu, Malaysia; tehsabariah@umt.edu.my; 3 Research Divisions, Association for Biodiversity Conservation and Research, Devine Colony, Balasore 756001, Odisha, India or anilchatterji@gmail.com (A.C.); ykmohanta@gmail.com (Y.K.M.); 4 Aquamarina Research Foundation, Dona Paula, Panaji 403004, Goa, India; 5 Centre of Excellence (CoE) for Bioresource Management and Energy Conservation Material Development, Fakir Mohan University, Balasore 756089, Odisha, India; parama.pondy@gmail.com; 6 Department of Food Technology and Biochemical Engineering, Faculty of Engineering and Technology, Jadavpur University, Jadavpur, Kolkata 700032, West Bengal, India; tanmays468@gmail.com; 7 Malda Polytechnic, West Bengal State Council of Technical Education, Govt. of West Bengal, Malda 732102, West Bengal, India; 8 School of Marine and Environmental Sciences, University Malaysia Terengganu, Kuala Nerus 21030, Terengganu, Malaysia; salwa.shahimi@umt.edu.my; 9 Forensic Science Programme, School of Health Sciences, Universiti Sains Malaysia, Health Campus, Kubang Kerian 16150, Kelantan, Malaysia; 10 School of Biotechnology, GIET University, Gunupur 765022, Odisha, India; dacharya249@gmail.com

**Keywords:** biopolymer, antioxidant, preservative, chitin, scavenging, natural product

## Abstract

Natural product extraction is ingenuity that permits the mass manufacturing of specific products in a cost-effective manner. With the aim of obtaining an alternative chitosan supply, the carapace of dead horseshoe crabs seemed feasible. This sparked an investigation of the structural changes and antioxidant capacity of horseshoe crab chitosan (HCH) by γ-irradiation using ^60^Co source. Chitosan was extracted from the horseshoe crab (*Tachypleus gigas*; Müller) carapace using heterogeneous chemical *N*-deacetylation of chitin, followed by the irradiation of HCH using ^60^Co at a dose-dependent rate of 10 kGy/hour. The average molecular weight was determined by the viscosimetric method. Regarding the chemical properties, the crystal-like structures obtained from γ-irradiated chitosan powders were determined using Fourier transfer infrared (FTIR) spectroscopy and X-ray diffraction (XRD) analyses. The change in chitosan structure was evident with dose-dependent rates between 10 and 20 kGy/hour. The antioxidant properties of horseshoe crab-derived chitosan were evaluated in vitro. The 20 kGy γ-irradiation applied to chitosan changed the structure and reduced the molecular weight, providing sufficient degradation for an increase in antioxidant activity. Our findings indicate that horseshoe crab chitosan can be employed for both scald-wound healing and long-term food preservation due to its buffer-like and radical ion scavenging ability.

## 1. Introduction

Chitin is the second most abundant biopolymer after cellulose, made of polysaccharides, and can be found in the cell wall of eukaryotes (i.e., fungi, yeast, protists, and diatoms). It is also an important substance for exoskeleton buildup in most invertebrates, including sponges, worms, molluscs, and arthropods. The commercial production of chitin is possible through crustacean (shrimp, crab, prawn, and crayfish) outer skeleton extractions [1,2,3,4]. In recent years, chitin applications have been scenic due to its remarkable biodegradability and biocompatibility properties, for example in cosmetics; pharmacy and medicine; biomaterial engineering; agriculture, textile, and paper industries; and environmental engineering [5,6,7,8,9]. Overall, chitin and its derivatives—mainly chitosan—are associated with more than 200 potential applications [10]. Generally, the industrial application of chitin involves alkaline hydrolysis, which is a procedure in which chitin is converted into a soluble polymer called chitosan. The polymer stimulates antimicrobial activity and is considered to be a significant crystalline homopolymer of *N*-acetyl glucosamine renewable sources [11].

The Indian horseshoe crab possesses a tough exoskeleton that offers protection from predators. To attain this feature, millions of tightly interwoven cellulose-like strands called chitin are orientated as tough and flexible glue and adhere to each other to form a shell. The chitin of horseshoe crab is favored for research and sought over other arthropod chitin because of its comparatively better purity [12]. Presently, the utilization of chemicals for food preparation is displaying an increasing demand. Therefore, food industries are turning their attention towards natural materials that preserve food from contamination from microbes. With the focus on this aspect, chitosan preparations are outlined considering their efficacy and safety, particularly when utilized as preservatives in the food industry. In this regard, chitosan seems effective for inhibiting microbial growth, promoting its use as a food preservative [13]. Since food and food-borne microbes are pH-sensitive, the ability of chitosan to regulate sodium ions in food makes it a suitable buffer that arrests microbial activity [14]. While γ-irradiation has the ability to modify chemical and physical properties of some polymeric materials [15], its ability to break polymeric chains promotes its use for improving the solubility of solvents [16,17] and stabilizing solutes [18,19] for antimicrobial [20] and antioxidant applications [21]. When γ-irradiation is applied to chitosan, the modification of chitosan molecules is thought to enhance the antimicrobial and antioxidant effects. However, knowledge on this form of application continues to be limited, apart from the application of chitosan comprised of different molecular weights for antioxidants [22] and antimicrobial [23,24] stimuli. We are also aware of the use of horseshoe crab chitosan as a bio-polymer, which has never been applied for the food industry [13]. Additionally, this form of chitosan has never been extracted, with the exception of our own findings [12]. In this study, the application of horseshoe crab chitosan against oxidative stress was conducted to identify the functional groups of γ-irradiated horseshoe crab chitosan. We evaluated the chitosan in terms of its molecular weight and determined its potential effect with regards to reducing the oxidative stress by defending the biotic or abiotic stress components.

## 2. Materials and Methods

### 2.1. Irradiation of Chitosanwith γ-Radiation

The carapace of dead horseshoe crabs was gathered, before crushing and grinding it into powder form. This was done to increase the surface area of exposure while converting chitin into chitosan by the following protocol [12,25]. After 24 h of storing the sample (4 °C in a conventional refrigerator) in low-density polyethylene bags, the horseshoe crab chitosan (HCH) was then separated into groups of different molecular weights using γ-irradiation treatment. In this step, ^60^Co was administered in a dose-dependent manner of 10 and 20 kGy and rate of 10 kGy/hour using Γ-PX-30 irradiation cells. Verification of this step employed the Fricke dosimetry system, whereas the control system used Red Perspex dosimeters. The samples were stored in the dark at room temperature after γ-irradiation and were subjected to various characterization tests to evaluate the effects of radiation with sample properties.

### 2.2. Determination of the Molecular Weight of Chitosan Using the Viscometric Method

For investigating the effect of irradiation on chitosan in terms of viscosity, the average molecular weight (M_v_) was obtained by determining the intrinsic viscosity of chitosan solutions in 0.3 M acetic acid/0.2 M sodium acetate at 25 ± 0.5 °C using a reported method [26]. The measurement started with the determination of solvent flow times (t_0_) and continued with flow times of samples (t_n_). From these flow times, the relative viscosity (η_r_), specific viscosity (η_sp_), and reduced viscosity (η_red_) were calculated. The value of the intrinsic viscosity (η) was calculated by extrapolating a plot of reduced viscosity against chitosan. Determinations were performed in triplicate using an Ubbelohde capillary viscometry technique and M_v_ was calculated using the Mark–Houwink–Sakurada equation [6,27], which provides the relationship between the intrinsic viscosity and molecular weight:

$$\left[\eta \right]=K{\left(M_{V}\right)}^{\alpha }$$

where [η] is the intrinsic viscosity, M_v_ is the molecular weight, and K and α are constant. K = 1.81 × 10^−3^ and α = 0.76.

### 2.3. Characterization of Chitosan Samples

Chitosan’s degree of deacetylation was determined using Fourier transform infrared spectroscopy (FTIR) (Thermo Nicolet™ 6700; Thermo Fisher Scientific, Waltham, MA, USA). In this step, the powder-form of chitosan was added with potassium bromide (KBr) *w*/*w* at 1:1, before being placed in a desiccator with silica beads to minimize moisture retention. It was incubated with 3 days oven drying (Memmert, Schwabach, Germany) before analysis using FTIR operating at a frequency of 4000–500 cm^−1^ and spectral range of 4 cm^−1^. The degree of deacetylation (DD) for chitosan was calculated as in Equation (1) [28].

(1) $$\mathrm{DD}=100-\;\left(\frac{\frac{A_{1655}}{A_{3450}}\;\times 100}{1.33}\right)$$

A_1655_ refers to the absorbance wavelength at 1655 cm^−1^ of the amide-I band. It was a measure of the *N*-acetyl group content. Meanwhile, A_3450_ refers to the absorbance wavelength at 3450 cm^−1^ of the hydroxyl band. The ratio was an internal standard used to correct the film thickness. The factor 1.33 refers to the ratio of A_1655_/A_3450_ for fully *N*-acetylated chitosan [29].

Separately, another batch of horseshoe crab chitosan was stored in a desiccator with silica beads overnight, before being mounted in its powder form onto a goniometer. An X-ray Panalytical Diffractometer (X’Pert Pro, Panalytical, Almelo, The Netherlands) operating with CuKα radiation (40 kV, 30 mA) at a wavelength λ of 1.54 nm continuously scanned the chitosan at a rate of 1°/minute from 5–45° over the 2θ range. Meanwhile, the chitosan stock was measured for its weight within the range of 5.2 and 5.4 mg, before being heated from 50 to 650 °C in the differential scanning colorimetric Pyris Diamond DSC (Perkin Elmer, Boston, MA, USA).

The thermogravimetry/differential thermal analysis (TG/DTA) was performed using a thermogravimetric analyzer (Pyris Diamond TG-DTA, PerkinElmer Instrument, Boston, MA, USA) with a heating rate of 10 °C/minute and dynamic synthetic atmospheric air with a flow of 100 mL/minute was set as a standard condition. In this study, the thermal behaviors of the irradiated horseshoe crab chitosan (HCH) were analyzed from the TG/DTA curves within the temperature range of 50–650 °C. At the initial time, the weight of the HCH was around 5.4 mg. TG/DTA was implemented to measure the change in weight of the sample when it was subjected to an isothermal temperature changed in a controlled way. On the other hand, chitosan comprised of different molecular weights (post-γ-irradiation) was placed onto a carbon tape and plated with gold using the sputter coater (POLARON-SC7620, Carbon Accessory, and Model-CA76, Quorum Technologies Ltd., Laughton, East Sussex, UK), before being observed through a scanning electron microscope (Carl Zeiss SMT, Oberkochen, Germany) operating at an accelerated voltage of 20 kV at a 4.0 kx magnification.

### 2.4. Antioxidant Activity

#### 2.4.1. DPPH Radical Scavenging Activity

The effect of γ-irradiated chitosan on DPPH radicals was investigated, using the modified method of the previous study [30]. Briefly, 100 µM stock was prepared by adding 39.4 mg DPPH(2,2-diphenyl-1-picrylhydrazyl) to 100 µL methanol and 1.0 mL of this solution was added to 4.0 mL test sample solution containing different concentrations of chitosan. The reaction mixture was shaken properly for 30 min in an incubation chamber at 37 °C and the absorbance of the resulting solution was measured at 517 nm to determine the final concentration. The DPPH scavenging activity of chitosan was calculated according to the following equation:

(2) $$\mathrm{DPPH}\;\mathrm{Scavenging}\;\left(\%\right)=\left(1-\left(\frac{A\;\mathrm{samples}\;517\;\mathrm{nm}}{A\;\mathrm{control}}\right)\right)\;\times 100.$$

#### 2.4.2. Superoxide Anion Radical Scavenging Assay

Chitosan was prepared in 0.2, 1.0, 2.0, 10, and 20 mg/mL aliquots as a control group. Conversely, 100 mM phosphate buffer (pH 7.4) was prepared, before 1 mL of this solution was added to4 mL horseshoe crab chitosan stock and the control solutions. Then, 50 µL of each chitosan solution was added to 50 µL of 300 µM nitro blue tetrazolium (NBT), 50 µL of 936 µM β-Nicotinamide Adenine Dinucleotide (NADH), and 50 µL of 120 µM phenazine methosulfate, before being incubated at room temperature for 5 min. It proceeded with an absorbance reading using a spectrophotometer set at a 560 nm wavelength. The scavenging capacity of the superoxide radical was estimated using adaptations of Robak and Gryglewski [31] by the following Equation (3).

(3) $$\mathrm{Superoxide}\;\mathrm{Scavenging}\;\left(\%\right)=\left(1-\left(\frac{A\;\mathrm{samples}\;517\;\mathrm{nm}}{A\;\mathrm{control}}\right)\right)\;\times 100$$

#### 2.4.3. Metal Ion Chelating Assay

The ferrous ion-chelating activity assay was conducted by adopting the method of Yen et al. [32], using spectrophotometer analysis at a 562 nm wavelength. The reaction mixture containing chitosan was added to 2 mM FeCl_2_ and 5 mM ferrozine, before being incubated for 10 min at room temperature. The EDTA was used as the positive control and the capacity of sulfated chitosan to chelate ferrous ions was calculated using the following Equation (4):

(4) $$\mathrm{Chelating}\;\mathrm{Effect}\;\left(\%\right)=\left(1-\left(\frac{A\;\mathrm{samples}\;517\;\mathrm{nm}}{A\;\mathrm{control}}\right)\right)\;\times 100.$$

#### 2.4.4. Total Reducing Power Ability

In the preparation of chitosan stock solutions, the aliquots of 1.0, 1.2, 1.4, 1.6, and 2.0 mg/mL were dissolved in 1% glacial acetic acid. The control was prepared using 1% glacial acetic acid solution as stock. All of the stock solutions were added to 0.5 mL 1% potassium ferricyanide in 0.5% acetic acid solution, before the addition of 0.2 M sodium phosphate buffer (pH 6.6) into a final volume of 1.5 mL. The reaction mixtures were incubated at 50 °C for 20 min. Subsequently, 0.5 mL of 10% trichloroacetic acid (TCA), 2 mL of distilled water, and 400 μL of 0.1% of ferric chloride were added into the mixtures, before centrifuging them at 3000 rpm for 10 min, and the absorbance was measured at 700 nm. An estimation of reducing the ability for chitosan was carried out by adopting the protocols of Oyaizu by following Equation (5) [33].

(5) $$\mathrm{Reducing}\;\mathrm{Ability}\;\left(\%\right)=\left(1-\left(\frac{A\;\mathrm{samples}\;517\;\mathrm{nm}}{A\;\mathrm{control}}\right)\right)\;\times 100$$

### 2.5. Statistical Analysis

Descriptive statistics (mean and standard deviation) and one-way analysis of variance (ANOVA) employed in SPSS v.17 (IBM Corp, Armonk, NY, USA) were used to evaluate the antioxidant ability of irradiated and non-irradiated horseshoe crab chitosan.

## 3. Results and Discussion

### 3.1. Production of Low Molecular Weight Chitosan

**Figure 1:**
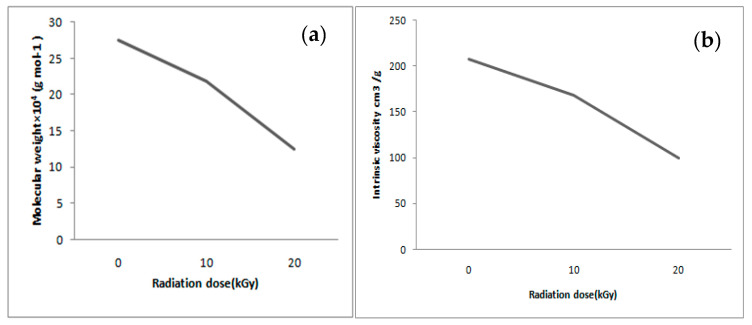
Effect of gamma radiation on the (**a**) Intrinsic viscosity and (**b**) molecular weight of chitosan.

The intrinsic viscosity and molecular weight of non-irradiated chitosan were 144.7 cm^3^/g and 187,128.42 gmol^−1^, respectively. However, irradiation caused a significant decrease in both the intrinsic viscosity and molecular weight of the chitosan. Figure 1a shows the decrease in the intrinsic viscosity of chitosan when applied with an irradiation dosage of 10 and 20 kGy. A direct relationship between the increase of the irradiation dose and the decrease of *M_W_* can be observed in Figure 1b. Usually, γ-exposure caused a scission reaction along with the 1–4 glycosidic bonds, which facilitated chitosan degradation and consequently reduced its molecular weight [6]. Probably, the break of the polymer chains is the prevailing process that occurred during the exposure of the polymer to the γ-ray, which also occurs when radiation is applied to marine-organism chitosan [34]. Moreover, the irradiation induces a depolymerization reaction that causes the scission of the molecule, which is prominent in smaller chitosan chains [35,36].

An identical approach was used in food preservation so that lower molecular weight chitosan (after γ-irradiation) promoted anti-oxidant activity [25]. However, industrial applications limit ^60^Co irradiation to a dose-dependent rate of 10 kGy/h, with a maximum dose of 50 kGy/h [25]. With reference to the 20 kGy doses of γ-irradiation used in the present study, both the covalent bond strength and chemical structure influenced the compaction and molecular weight of chitosan. In this case, the use of chitosan from horseshoe crabs indefinitely reduced the industrial costs, without compromising functional groups, when intended for food preservation.

### 3.2. Characterization of Horseshoe Crab Chitosan

The degree of horseshoe crab chitosan deacetylation pertains to the quantity of glucosamine within the biopolymer chains during the transition of chitosan from chitin. While the bands 1221 and 1505 cm^−1^ were adopted as a reference and non-irradiated chitosan produced an 83.90% degree of deacetylation, whereas for 10 and 20 kGy irradiated chitosan, the value was 85% and 86%, respectively. Taking into account the reduced molecular weight of chitosan and the inversely increasing degree of deacetylation after irradiation, altering the chitosan crystallinity and viscosity amounts to degradation [31] that favors its use in the food industry [25]. The availability of low molecular weight chitosan favors the manufacturing of low viscosity liquids and omits the need for demineralization processes (reducing the manufacturing costs). Considering the intrinsic viscosity, for irradiated chitosan at 10 and 20 kGy, the valueswere 160.00 and 98.80 cP, respectively, which are associated with a low range of viscosity if compared to industrial paramount [30,31,37,38].

**Figure 2:**
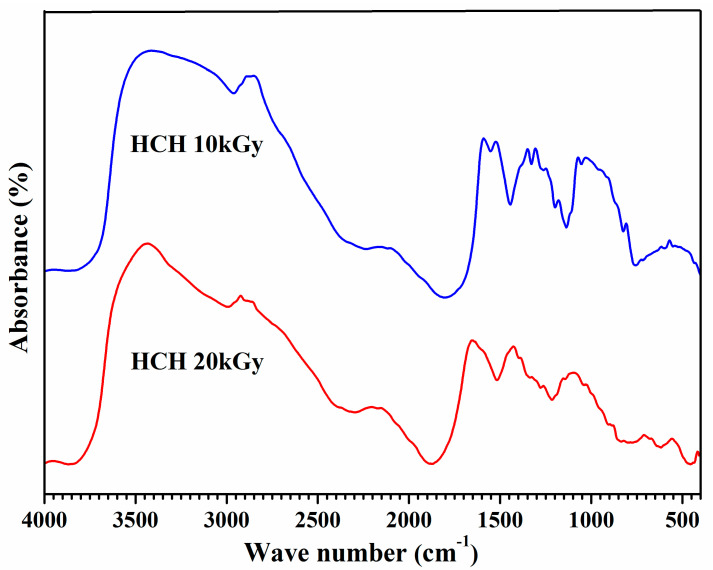
Fourier transfer infrared (FTIR) spectra of horseshoe crab chitosan (HCH) irradiated at 10 and 20 kGy.

Hydroxyl (–OH) stretching bands of horseshoe crab chitosan were 3435.81 cm^−1^ and, for the C–H group, they were 2995 and 2988.94 cm^−1^ after the 10 and 20 kGy irradiation, respectively (Figure 2).

Chitosan’s C=O stretching band (pyranose ring) was 1878 cm^−1^, its NH group stretching bands occurred at 1512.65 cm^−1^, and the NHCO amine complexes produced bands of 1221 and 1214.20 cm^−1^. While identical stretch bands were also retrieved in past findings [39], conformities to identical samples suggest that irradiation up to 20 kGy through a ^60^Co source breaks double bonds to reduce the molecular weight of horseshoe crab chitosan. However, –NH groups were less affected by the γ-irradiation (decreased FTIR transmittance band) and appeared to have a close resemblance to non-irradiated chitosan [12]. Again, chitosan sources were crucial, particularly for obtaining the optimal degree of deacetylation after γ-irradiation, because it was 75% for the present study and much lower for non-arthropod sources [40].

**Figure 3:**
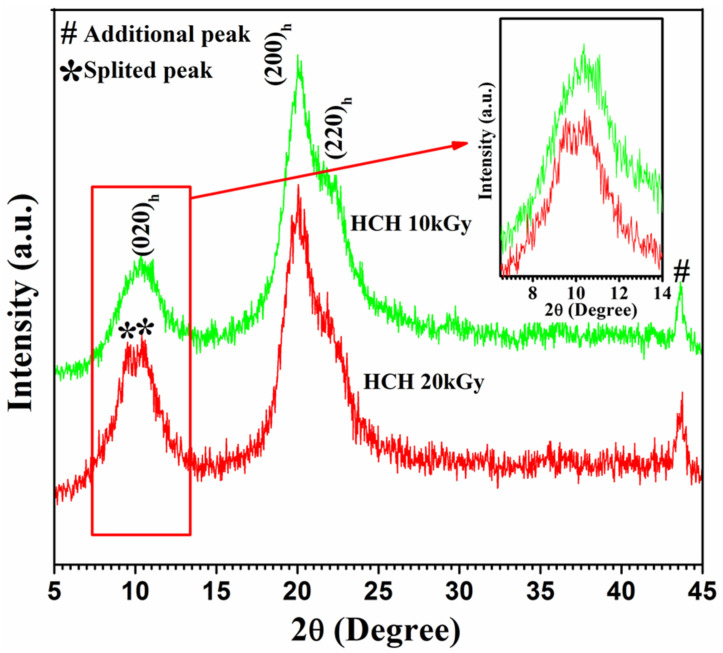
X-ray diffraction (XRD) patterns of HCH irradiated at 10 and 20 kGy.

**Figure 4:**
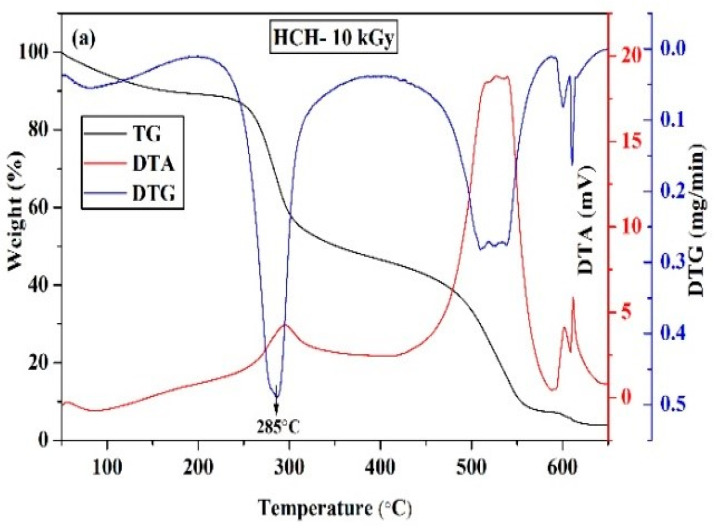

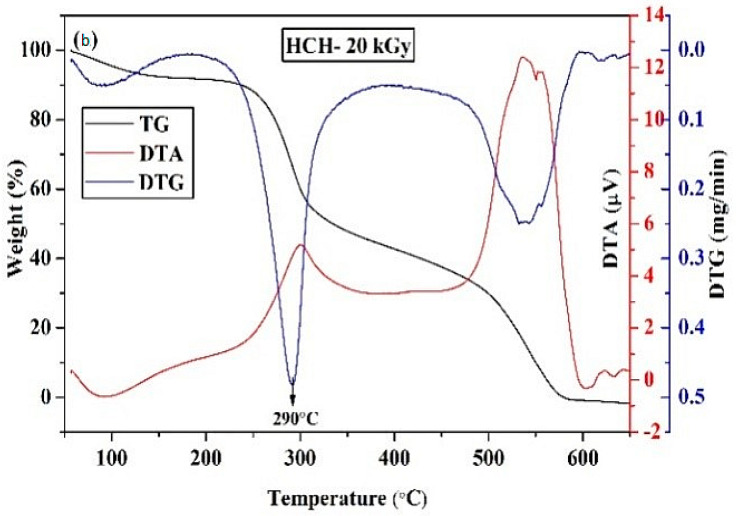
Thermogravimetry (TG), DTG, and differential thermal analysis (DTA) curves of irradiated chitosan: (**a**)HCH-10 kGy and (**b**) HCH-20 kGy.

Peculiarly, XRD patterns for 10 kGy γ-ray-irradiated chitosan peaks at 2θ = 10.5°, 20.04°, and 21.9° with reflections were 20, 200, and 220 h, respectively. (Figure 3), which was identical to non-irradiated chitosan [12,13,14,15,16,17,18,19,20,21,22,23,24,25,26,27,28,29,30,31]. However, when the 20 kGy γ-ray was used, an additional peak developed at 2θ = 10.5°. This was an indication that increased γ-irradiation splits the peak at 2θ = 10.5°into two separate attributes because of chitosan crystalline structure manipulation. When thermal manifestation (50 to 650 °C) was applied to chitosan after γ-irradiation, it was discovered that degradation and decomposition occurred first, before the residue remained (Figure 4). In this sense, heating chitosan at 100 °C causes water evaporation and proceeds with endothermic dehydration [41].

Moreover, heat exposure between 100 to 150 °Ccaused thermal degradation and complete evaporation before depolymerization when the temperature reached 270 °C (Figure 4a,b). The 60% weight loss of chitosan at 300 °C (optimal decomposition temperature range of 303–306 °C) indicates that amino polymers were less stable than *N*-acyl bonds. The effects of γ-irradiation on altering the chitosan crystalline structure wore out during degradation when chitosan was exposed to a temperature of 430 °C. Since irradiated chitosan has better thermal stability than non-irradiated chitosan [12], the present findings suggest that *N*-acyl bonds were reinforced with the 10 and 20 kGy γ-irradiation.

**Figure 5:**
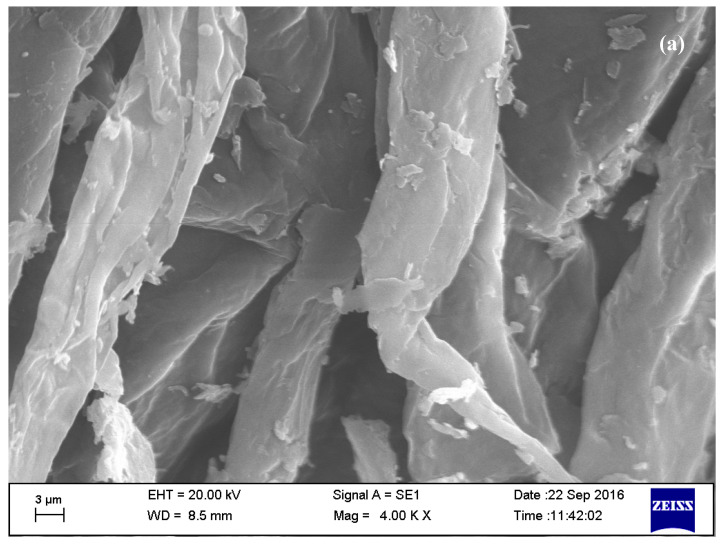

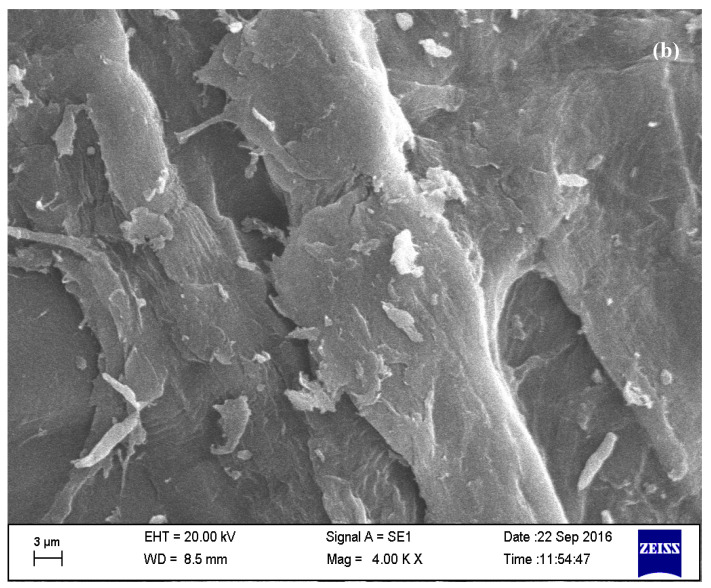
SEM micrographs of irradiated chitosan: (**a**) HCH-10 kGy and (**b**) HCH-20 kGy.

The SEM micrographs of the irradiated chitosan surface (Figure 5a,b) indicated folding and layering, while non-irradiated chitosan has a fiber-like surface [12]. The difference in the morphological appearance of the irradiated and non-irradiated chitosan might be attributed to the breaking of bonds during irradiation.

### 3.3. Antioxidant Activity

**Figure 6:**
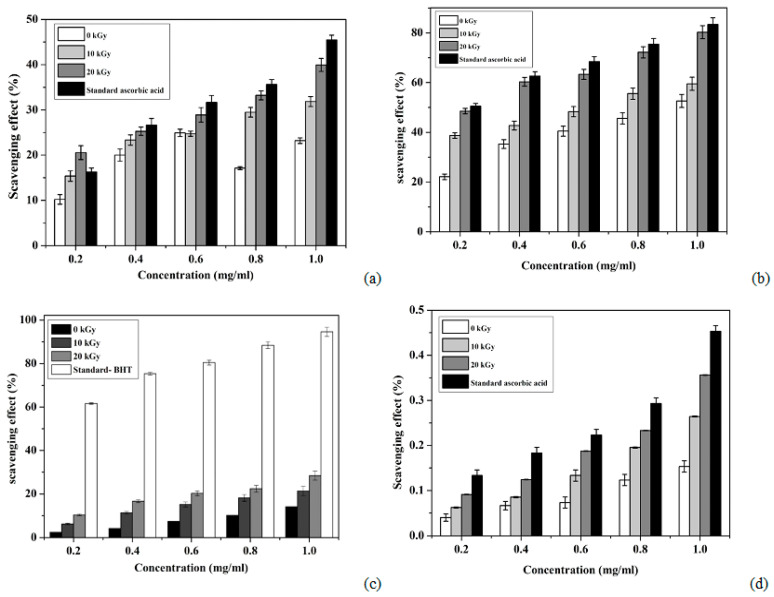
Antioxidant activity of horseshoe crab chitosan after different irradiation exposure. (**a**) Scavenging effects of horseshoe crab chitosan with different doses and ascorbic acid on DPPH radicals. Each value is presented as the mean ± SE (*n* = 3) and values were significantly different at *p* < 0.05. (**b**) Scavenging effects of horseshoe crab chitosan with different doses (DDC) and ascorbic acid on superoxide anion radicals. Each value represents the mean ± SE (*n* = 3) and values were significantly different at *p* < 0.05. (**c**) Chelating effects of horseshoe crab chitosan with different doses and EDTA on ferrous ions. Each value is presented as the mean ± SE (*n* = 3) and values were significantly different at *p* < 0.05. (**d**) The total reducing power ability of Butylated hydroxytoluene and the different doses of chitosan from (DDC) horseshoe crab. Data are presented as the mean ± S.E. (*n* = 3) and were significantly different at *p* < 0.05.

After exposure to DPPH, the superoxide radical scavenging ability followed the order of ascorbic acid (45.48 ± 1.05%) > 20 kGy (39.95 ± 1.43%) > 10 kGy (31.86 ± 1.09%) > 0 kGy (23.21 ± 0.62%) (Figure 6a), with chitosan exhibiting some antioxidant ability. A 37.33% (for 10 kGy) and 72.12% (20 kGy) increase was witnessed for DPPH activity with respect to the untreated sample (0 kGy). It has been reported that the antioxidant activity of chitosan increases with the increase of irradiation doses and decreases the *M_W_* [21]. It was reported that one of the mechanisms of chitosan is related to its scavenging activity, which can respond with free radical leftover free –NH_2_ groups to form stable molecules and the –NH_2_ groups can form ammonium groups (${\mathrm{NH}}_{3}^{+}$) by capturing a hydronium ion from the solution [32].

The reconfigured horseshoe crab chitosan can be functionalized to reduce oxidative stress, as well as employed externally for scald-wound healing [42,43,44,45]. An identical trend was also achieved for the superoxide anion scavenging activity (Figure 6b); however, the margin of differences for this activity was small, as indicated by the ascorbic acid (83.42 ± 0.03 mg/mL) and 20 kGy (80.26 ± 0.02 mg/mL) exposures to chitosan. Only a 3.93% enhancement in the superoxide anion scavenging activity was observed for the 20 kGy irradiated product. On the contrary to DPPH, superoxide radicals are naturally generated reactive oxygen species within the cell matrix, so the superoxide anion scavenging activity actually mimics the actual condition within the human body and is thus able to give an overview of how the product will act in an in-vivo condition [31]. Comparatively, fungal chitosan displayed a radical scavenging ability of 2.84–5.35% [38], whereas for Shiitake stipes and crab shell, it was lower, at 2.84% [46,47], if compared to *Portunus pelagicus* and *Podophthalmus vigil* crab shell, which showed 4.64–5.23% [38] and 5.56% values for their chitosan [48].

Horseshoe crab chitosan appeared to be more than 40 times more effective than marine crab shells, and the addition of NaOH during *N*-deacetylation enhanced its radical scavenging ability. While the radical scavenging ability was inversely correlated with the chitosan molecular weight, the hydroxyl and amine groups in the chitosan molecule were responsible for the radical scavenging activities. Therefore, more highly deacetylated chitosan would have higher radical scavenging activities [48,49]. Unfortunately, acidic conditions (EDTA) made the amine groups of horseshoe crab chitosan inert and less effective for anti-oxidant activity. Despite this, chitosan with a reduced degree of acetylation has its amino acid readily available for metal adsorption [50]. When tested independently, EDTA effectively chelates ferrous ions (94.62 ± 0.02%) [51]. However, decreasing the molecular weight of horseshoe crab chitosan after 0 kGy (14.16 ± 0.03%), 10 kGy (21.35 ± 0.04%), and 20 kGy (28.43 ± 0.02%) γ-exposure (Figure 6c) showed increasing activity against radical scavenging. A 50.77%- and 1.07%-fold increase in the ferrous ion chelating activity was recorded for the 10 and 20 kGy irradiated products, respectively.

Additionally, the ferrous-ion chelation rate was proportional to the concentration of chitosan used, and an identical observation was also seen with fungal chitosan [46]. The molecules of chitosan may act as a chelating ligand at the second and third carbon atoms of the chitosan monomer and form chelates with Fe^2+^. Irradiation may cause a lower *M_W_* compound, which in turn will expose more amine and hydroxyl groups, and these play a pivotal role in determining the ferrous-ion chelation rate. In this regard, selection of the γ-irradiation limit (10–20 kGy) made the horseshoe crab chitosan effective for hydroxyl (50.47 ± 0.1%) and superoxide (52.65 ± 0.04%–80.26 ± 0.02%) radical scavenging if compared with *Podophthalmus vigil* shell chitosan (6.25% and 1.21–7.74%, respectively) [48]. When tested against diethyldithiocarbamate, the order for radical scavenging was butylated hydroxytoluene (0.46 ± 0.01%) > 20 kGy (0.36 ± 0.001%) > 10 kGy (0.26 ± 0.001%) > 0 kGy (0.15 ± 0.01%) (Figure 6d). Therefore, from the results, it is obvious that there is a 2.40- and 1.73-fold increase in the total reducing power of the chitosan produced at 20 and 10 kGy, respectively, in comparison to the sample unexposed to any radiation. Our findings clearly showed that the antioxidant property of horseshoe crab chitosan was inversely related to its molecular weight. This might be due to the chitosan with larger *M_W_* having a more compact structure, which results in stronger intramolecular hydrogen bonds and restricts the hydroxyl and amine groups from reacting with radicals or metal ions. Conversely, the horseshoe crab chitosan reaction rate against free radicals increases with the γ-irradiation and dosage concentration; however, it also has an overdose limit that should not exceed the 1:1 *w*/*w* ratio. While chitosan from horseshoe crab carapace may seem beneficial to the biomedical industry due to its negligible use in India, this is not the situation in other Asian countries, where *T. gigas* was discovered [52,53,54,55,56], and another form of chitosan that originates from Orthoptera (*Calliptamus barbarous* and *Oedaleus decorus*) was proposed [57]. Regarding this, chitin and chitosan extraction from invasive or pest species may be useful for controlling over-breeding populations because their bioactive compounds are replenishable (large population size) and reducing production costs in food manufacturing industries. The vast number of abandoned carapaces usually causes environmental issues and marine ecology pollution. Alternatively, this waste can be used as an economic source of chitosan and its derivatives. The characteristics of the horseshoe crab chitosan produced in our study were according to the commercial standard.

## 4. Conclusions

The horseshoe crab chitosan powder samples (HCH) were successfully subjected to the γ-irradiation of 10 and 20 kGy doses using a ^60^Co source. The γ-irradiated chitosan powder samples were characterized by FTIR, XRD, SEM, and TG/DTA and underwent a change in structure at different doses, with a reduction of the molecular weight. The antioxidant activity of horseshoe crab chitosan was compared for irradiated and non-irradiated forms when applied against antioxidizing agents, such as DPPH, superoxide anions, ferrous ions, and diethyldithiocarbamate. While horseshoe crab chitosan was reconfigured using γ-exposure, it reduced in molecular weight and appeared with active amino acid groups. The addition of NaOH increased the rate of chitosan deacetylation, which is an important criterion for enhancing irradiated chitosan’s ability against radicals.
